# Do changes in red blood cell deformability in patients with septic shock correlate with changes in SOFA scores?

**DOI:** 10.1186/cc11949

**Published:** 2013-03-19

**Authors:** T Clark, S Jewell, M Sair, P Petrov, P Winlove

**Affiliations:** 1 Derriford Hospital, Plymouth, UK; 2 University of Exeter, UK

## Introduction

Traditional whole blood experiments suggest that sepsis causes abnormal red blood cell (RBC) deformability. To investigate this at the cellular level, we employed a novel biophysical method to observe individual RBC membrane mechanics in patients with septic shock.

## Methods

We collected blood samples from patients with septic shock until either death or day 5 of admission. Thermal influctuations of individual RBCs were recorded allowing a complete analysis of RBC shape variation over time. Mean elasticity of the cell membrane was then quantified for each sample collected.

## Results

We recruited nine patients with septic shock. Table 1 shows mean RBC thermal fluctuation and SOFA scores.

**Table 1:** Mean RBC fluctuation (daily SOFA score)

Day	A	B	C	D	E	F	G	H	I
1	4.8 (10)	5.2 (9)	-	4.8 (12)	4.6 (16)	4.9 (13)	5.1 (16)	4.6 (18)	5.1 (15)
2	4.9 (9)	5.0 (10)	4.6 (13)	5.1 (11)	4.8 (17)	5.1 (13)	5.0 (16)	4.9 (19)	- (16)
3	4.0 (6)	5.1 (9)	4.7 (12)	4.8 (11)	4.9 (18)	4.8 (13)	5.0 (16)	4.7 (21)	5.3 (16)
4	-	4.8 (7)	4.6 (11)	5.0 (9)	5.9 (19)	-	- (18)	-	5.0 (15)
5	-	-	-	5.1 (6)	5.2 (18)	-	- (19)	-	5.0 (10)

## Conclusion

RBC thermal fluctuation analysis allows variations in RBC elasticity during sepsis to be quantified at a cellular level. We could not identify any specific trend between sepsis severity and erythrocyte elasticity. Cells demonstrated both increases and decreases in fluctuation independent of SOFA score. This is contrary to current evidence that suggests RBC deformability is reduced during sepsis.
